# Recurrence risk in small, node-negative, early breast cancer: a multicenter retrospective analysis

**DOI:** 10.1007/s00432-013-1388-2

**Published:** 2013-02-15

**Authors:** T. Gamucci, A. Vaccaro, F. Ciancola, L. Pizzuti, I. Sperduti, L. Moscetti, F. Longo, M. A. Fabbri, M. A. Giampaolo, L. Mentuccia, L. Di Lauro, P. Vici

**Affiliations:** 1 Medical Oncology Unit, ASL Frosinone, Via Armando Fabi, 03100 Frosinone, Italy; 2 Medical Oncology B Division, Regina Elena National Cancer Institute, Via Elio Chianesi 53, 00144 Rome, Italy; 3 Department of Biostatistics, Regina Elena National Cancer Institute, Via Elio Chianesi 53, 00144 Rome, Italy; 4 Medical Oncology Unit, Belcolle Hospital, Strada Sammartinese 1, 01100 Viterbo, Italy; 5 Medical Oncology Unit A, La Sapienza University of Rome, Viale del Policlinico 161, 00161 Rome, Italy

**Keywords:** Small breast cancer, Node-negative, Recurrence risk, Prognostic biological factors

## Abstract

**Purpose:**

Recurrences and deaths are known to occur, even if less frequently, in small, node-negative breast cancer patients, and decision on adjuvant treatments remains controversial. In the present analysis, we evaluate recurrence risk in patients with pT1 a, b, c, node-negative, breast cancer, accordingly with some prognostic biological factors.

**Methods:**

We retrospectively evaluated 900 node-negative patients (pT1a, b, c) surgery treated between 2000 and 2009 in four Italian oncologic centers. We defined 3 different cohorts: ER positive (ER+); Her-2 positive (Her-2+); and triple negative (TN).

**Results:**

pT1a was seen in 7.6% of patients, 37.7 % pT1b, 54.8 % pT1c. Concerning the 3 different cohorts, 58.2 % were ER+; 10.8 % were Her-2+; 8.2 % were TN. Overall, chemotherapy was given to 3.0 %, 27.2 %, 69.8 % of pT1a, b, c, respectively, and to 22.7 %, 58.8 %, 68.9 % of ER+, Her-2+, TN subgroups. At a median follow-up of 67 months, 5-year DFS was 96.3 %, 89.2 %, 89.4 % in pT1a, b, c, respectively (100 %, 93.6 %, 89.8 % in ER+; 100 %, 78.7 %, 85.0 % in Her-2+; 100 %, 76.8 %, 85.2 % in TN) (*p* = ns). At multivariate analysis, histologic grade and Ki-67 resulted independent prognostic factors. Overall, 5-year OS was 98 %, without differences among pT1a, b, c, or among the 3 cohorts.

**Conclusions:**

Overall, 5-year DFS was very favorable in this series of small, node-negative breast cancers, but Her-2+ and TN cohorts have a higher recurrence rate than ER+ cohort (*p* < 0.0001); pT1c, but also pT1b, in Her-2+ and TN subgroups, have a worse outcome, and effective chemotherapy treatment should be considered in these unfavorable subgroups.

## Introduction

Despite the recent emergence of new prognostic factors and gene expression profiling, tumor size, together with axillary nodal status, is still considered a key factor in breast cancer biology and has important therapeutic implications. Recent knowledge suggest that breast cancer is a heterogeneous disease, with some characteristics, such as Her-2, hormonal receptors, and other variables being a fundamental and critical determinants of clinical outcome, with high prognostic value (Foulkes et al. 2010). Moreover, the incidence of small (≤1 cm) invasive breast cancers without axillary lymph nodes involvement has been increasing because of broader use of mammographic screening and can be considered an emerging topic (Benson et al. 2009). We all know that, particularly in some subtypes of breast cancer, small tumors might harbor cells inherently aggressive and are likely to metastasize, with no relationship between cancer cells number (size) and metastatic potential. In the last years, TNM staging, Her-2 and hormonal receptor status, tumor grading, and lymphovascular invasion have been routinely considered in stage I breast cancer treatment decision, but, unfortunately, there is no general agreement on the magnitude of benefit from adjuvant systemic treatments, and clear guidelines are lacking in this patient subset (McArthur et al. 2011; Kwon et al. 2010; Ho et al. 2012; Amar et al. 2010).

Many studies suggest that Her-2 positivity is an independent predictor of recurrence and death (Buzdar et al. 2005), and five randomized phase III trials reported significant advantages in disease free survival (DFS) and overall survival (OS) with trastuzumab plus chemotherapy over chemotherapy alone in Her-2 positive early breast cancer, but the majority of tumors were node-positive and larger than 1 cm (Joensuu et al. 2006; Piccart-Gebhart et al. 2005; Romond et al. 2005; Slamon et al. 2011; Perez et al. 2011); moreover, available data in 5 and 10-year recurrence rate in Her-2 positive, node-negative, small (≤1 cm) tumors are limited (Viani et al. 2007). Also for triple negative breast cancer evidences suggest greater recurrence risk even in “low-risk” category, being the triple negative phenotype an independent marker of poor prognosis, but definite guidelines concerning chemotherapy treatment exist only for pT1cN0 stage, and the decision to provide or not systemic adjuvant chemotherapy in tumors smaller than 1 cm is still a subject of controversy (NCCN Clinical Practice 2012). A number of “old” retrospective series on small node-negative breast cancer natural history gave heterogeneous relapse-free survival (RFS) rates, ranging from 87 % at 5 years to more than 90 % at 10 years (Hanrahan et al. 2007).

In the last few years, many retrospective reviews of T1N0 breast cancer patients have been published to determine the natural history of untreated tumors and the impact of systemic treatments on this subset of patients (McArthur et al. 2011; Amar et al. 2010; Curigliano et al. 2009; Cancello et al. 2011; Lai et al. 2011; Garassino et al. 2009; Kaplan et al. 2009; Gonzalez-Angulo et al. 2009), with in some cases contradictory results, and maintaining uncertainties concerning biology, prognosis, and optimal management of small node-negative breast cancers; moreover, some of these studies suffered from small sample size, low number of events, different end points, old methodologies to define biological features, lacking of data, short follow-up, and not all results available as full paper.

The aim of the present multicenter retrospective study was to evaluate recurrence risk in patients diagnosed with pT1a, b, c, node-negative, breast cancer, accordingly to some immunohistochemically defined prognostic biological factors.

## Patients and methods

We retrospectively identified pT1N0 breast cancer patients who received surgery between 2000 and 2009 in four Italian oncologic centers. Tumor staging was based on TNM system (WHO). Eligibility criteria included complete surgical resection, histologic diagnosis of invasive ductal or lobular carcinoma of 2 cm or less, and no lymph node metastasis; patients with history of previous breast cancer or other malignancies were excluded. Patients who received neoadjuvant chemotherapy or with ductal or lobular carcinoma in situ or microinvasive breast cancer were excluded from the analysis. All patients received locoregional treatment, with breast conservative surgery or mastectomy with axillary lymph node dissection or sentinel node biopsy, and postoperative radiotherapy whenever appropriate; systemic adjuvant treatment was given accordingly to stage and biological characteristics.

Pathologic data were obtained by collecting and reviewing pathology reports on tumor size, histologic subtype, modified Bloom-Richardson histologic grade. Immunohistochemical data for hormonal receptors, Her-2, p53, Ki-67 were also collected. Significant expression of hormonal receptors was defined as estrogen and/or progesterone receptor immunostaining of more than 10 % of invasive cells. Her-2 receptor expression level 3+ staining by immunohistochemistry (DAKO Herceptest) was considered positive, and FISH (fluorescence in situ hybridization), or CISH (chromogenic in situ hybridization), or SISH (silver in situ hybridization) evaluation was performed in Her-2 2+ staining. The Ki-67 threshold value was 15 %. Histologic grade and biological features were evaluated on the invasive component of the tumor.

Patients were divided into three groups according to their combined hormonal receptor and Her-2 status: ER and/or PgR positive, with Her-2 negative (ER+); Her-2 overexpressed or amplified (Her-2+); triple negative (TN). Survival analysis was performed for patients surgery treated until December 2008 (773 patients).

### Statistical analysis

Disease free survival was calculated as the time from surgery to recurrent disease (local, regional or distant site); for patients who remained alive and disease free, data were censored at the last follow-up. OS was measured from date of surgery to date of death of any cause, or latest follow-up. Contralateral breast cancer was included in recurrent disease.

The associations between variables were tested by the Pearson’s chi-square test or Fisher’s exact test, when appropriate. Kaplan–Meier method was used to estimate survival curves and differences among them assessed by the logrank test. The hazard ratio (HR) and the 95 % confidence intervals (95 % CI) were estimated for each variable by the Cox univariate model. A multivariate Cox proportional hazard model was also developed by stepwise regression (forward selection); enter limit and remove limit were *p* = 0.10 and *p* = 0.15, respectively. The assessment of interaction among significant variables was taken into account when developing the multivariate model. The SPSS software (SPSS version 19.0, SPSS Inc., Chicago, Illinois, USA) was used for all statistical evaluations.

Approval for the study was obtained from all institutional review boards.

## Results

From January 1999 to December 2010, we identified a total of 900 women with surgery-treated pT1N0 invasive breast cancer from 4 Italian oncologic centers. The distribution of patients in the three cohorts was as follows: 524 (58.2 %) ER+; 97 (10.8 %) Her-2+; 74 (8.2 %) TN. The remaining 205 tumors were lacking of complete data for being included in the three cohorts but have been analyzed for other parameters.

Main patient and tumor characteristics on the whole series of 900 patients are reported on Table 1. As tumor size concerns, pT1a, b, c were 7.6%, 37.7 %, 54.8 %, respectively; ER and/or PgR were positive in 83.7 % of the tumors; Her-2 was positive in 10.8 % (70 % also ER+) of the tumors; Ki-67 was >15 % in 28 % of the tumors; ductal, lobular, other histotypes were recorded in 87.4 %, 6.3 %, 6.3 % of the tumors. High histologic grade (G3) was recorded in 19.3 % of the tumors, closely related to Her-2+ (47.7 %) or TN subtypes (52.9 %), while it was rarely evidenced in ER+ cohort (12.6 %) (*p* < 0.0001). Her-2+ and TN subtypes were more frequent in young (<35 years) patients (3.1 % and 6.8 % versus 1.1 % in ER+ subgroup, *p* 0.004).

**Table 1:** Patient characteristics at baseline

Characteristic (*N* = 900)	*N* (%)
Age	
Median	58
Range	21–86
Menopausal status	
Pre	249 (27.7)
Post	651 (72.3)
Histology	
Ductal	786 (87.4)
Lobular	57 (6.3)
Other	57 (6.3)
Tumor size	
T1a	68 (7.6)
T1b	339 (37.6)
T1c	493 (54.8)
Grading	
G1	179 (19.9)
G2	450 (50.0)
G3	174 (19.3)
Unknown	97 (10.8)
Ki67	
<15 %	478 (53.1)
>15 %	252 (28.0)
Unknown	170 (18.9)
Hormone receptor status	
Positive	753 (83.7)
Negative	136 (15.1)
Unknown	11 (1.2)
HER-2/neu status	
Overexpressed or amplified	97 (10.8)
Negative	599 (66.5)
Unknown	204 (22.7)

Breast conservative surgery was performed in 736 patients (81.8 %). Postoperative radiotherapy was given to 79.4 % of the patients. The rate of breast conservative surgery and radiotherapy not significantly differed among ER+, Her-2+, TN subgroups.

Adjuvant endocrine treatment (tamoxifen or aromatase inhibitors) was given to 97.1  % of the patients in the ER+ and to 73.2 % of the patients in the Her-2+ cohort.

**Fig. 1:**
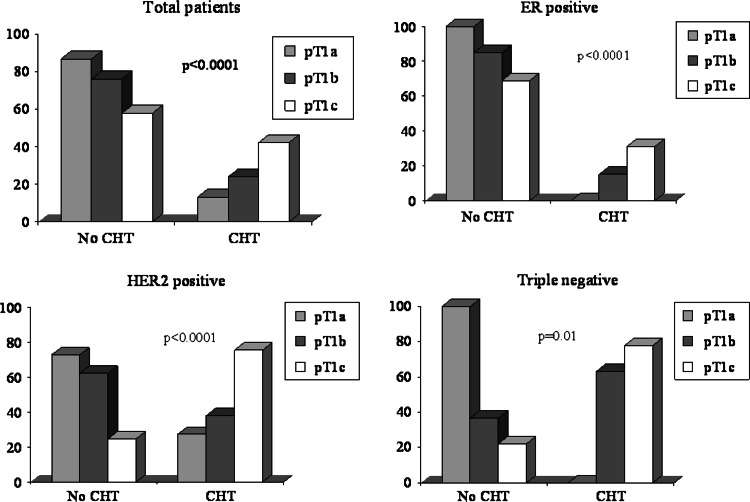
Adjuvant chemotherapy (CHT)

Overall, adjuvant chemotherapy has been delivered to 3.0 %, 27.2 %, 69.8 % of pT1a, b, c, patients, respectively. In ER+ cohort, chemotherapy (70.6 % anthracycline-containing regimens, 10.9 % taxane-based regimens, 18.5 % other regimens) has been administered to 119 (22.7 %) patients (0 %, 26.1 %, 73.9 % of pT1a, b, c tumors, respectively). In Her-2+ subset, chemotherapy alone has been delivered to 26 (26.8 %) patients (0 %, 30.8 %, 69.2 % of pT1a, b, c tumors), whereas chemotherapy plus trastuzumab was given to 31 (32 %) patients (9.7 %, 9.7 %, 80.6 % of pT1a, b, c tumors). In the TN cohort, chemotherapy has been administered in 51 (68.9 %) patients (0 %/37.3 %/62.7 % of pT1a, b, c tumors, respectively) (Fig. 1).

Ki-67 was higher than 15 % in 62.3 % of TN and in 59.5 % of Her-2+ tumors, whereas in ER+ subtype, this issue was recorded in 26.8 % of the tumors (*p* < 0.0001). Grade 3 was found in 52.9 % of TN, 47.7 % of Her-2+, and only in 12.6 % of ER+ tumors (*p* < 0.0001).

On the whole cohort of patients, with a median follow-up of 67 months (range, 10–279), the 5-year DFS was very favorable, being 96.3 %, 89.2 %, 89.4 %, in pT1a, b, c, respectively (*p* 0.35). In ER+ subtype, 5-year DFS was 100 %, 93.6 %, 89.8 % in pT1a, b, c tumors (*p* 0.12). In Her-2+ cohort, 5-year DFS was 100 %, 78.7 %, 85.0 % in pT1a, b, c, respectively (*p* 0.40), whereas in TN subtype, these issues were 100 %, 76.8 %, 85.2 %, in pT1a, b, c tumors (*p* 0.65).

**Fig. 2:**
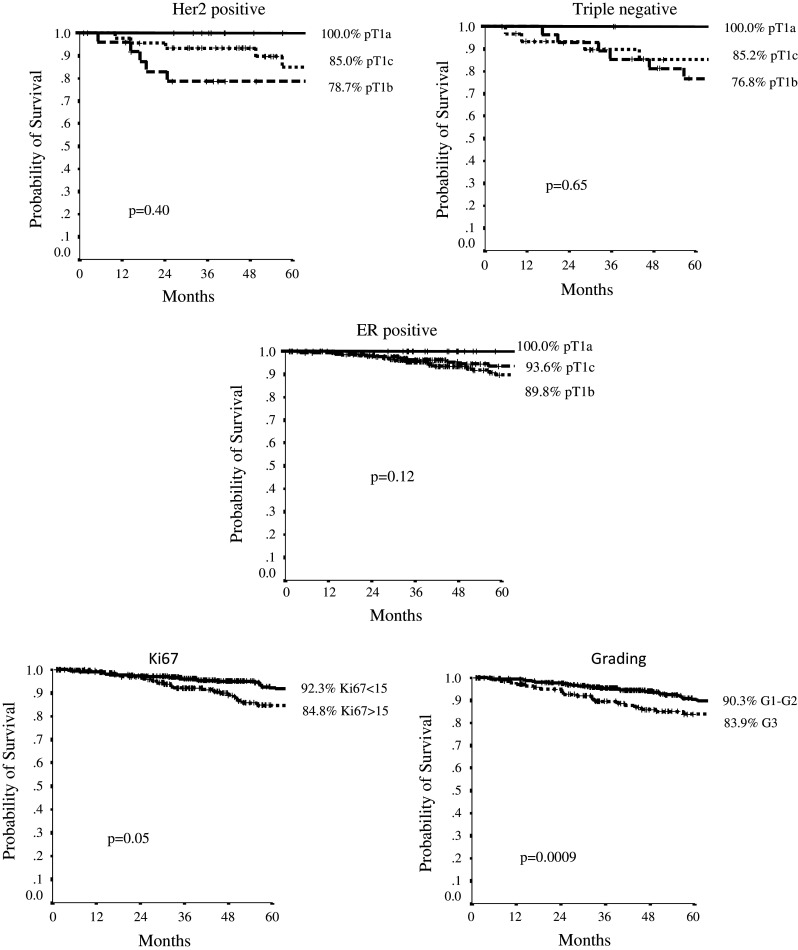
5-year DFS according to cohorts and significant parameters at Cox analysis

Figure 2 reports 5-year DFS curves according to Her-2, TN, ER status, histologic grading (G1-2 vs G3: 90.3 % vs 83.9 %, *p* 0.0009), and Ki-67 (≤15 vs >15 %: 92.3 % vs 84.8 %, *p* 0.05).

Overall, patients with small, node-negative breast cancer have an excellent prognosis, but, as expected, Her-2+ and TN appear to have a higher recurrence rate than ER+ cohort, being 13.4 % and 27 % versus 9.6 %, *p* < 0.0001. The subgroups of pT1b in the Her-2+ and TN cohorts showed a slightly higher, even if not significant for the small sample size, recurrence rate than larger tumors (pT1c) (5-year DFS, 77.1 % vs. 85.6 %, respectively, *p* 0.31); this may be presumably related to the higher percentage of patients treated with chemotherapy in the pT1c groups. We cannot discern in the Her-2+ cohort how much of the benefit can be ascribed specifically to trastuzumab versus chemotherapy administration, giving the small sample size.

We performed a Cox multivariate analysis, including significant clinical and biological features at univariate analysis, and histologic grading (HR 2.58, 95 % CI, 1.26–5.25, *p* 0.009) and Ki-67 (HR 2.11, 95 % CI, 1.07–4.15, *p* 0.03) were confirmed to be independent prognostic factors (Table 2).

**Table 2:** Analysis of prognostic factors for DFS with Cox regression model

Variables		Univariate analysis		Multivariate analysis	
		HR (95 % CI)	*p* value	HR (95 % CI)	*p* value
Age	>58 versus ≤58	1.22 (0.83–1.79)	0.31		
T stage	>1 cm versus <1 cm	1.60 (0.94–2.71)	0.08		
T stage	1a versus 1b versus 1c	NA	0.14		
	1b versus 1a	3.54 (0.47–26.4)	0.22		
	1c versus 1a	5.05 (0.69–36.73)	0.11		
	1b versus 1c	0.70 (0.41–1.20)	0.38		
Grading	3 versus 12	2.92 (1.69–5.04)	<0.0001	2.58 (1.26–5.25)	0.009
Surgery	mast versus quadr	1.49 (0.98–2.26)	0.6		
Cohorts	A versus B versus C	NA	0.38		
	B versus A	1.49 (0.65–3.45)	0.35		
	C versus A	1.63 (0.74–3.60)	0.23		
	B versus C	0.92 (0.33–2.53)	0.86		
Ki67	>15 versus <15	2.68 (1.46–4.92)	0.001	2.11 (1.07–4.15)	0.03

*NA* not assessed. HR and CI can only be calculated for two variables

A: ER pos

B: *TN*

C: HER2 pos

*HR* hazard risk

The 5-year OS was 98 %, without differences among tumor size (pT1a, b, c: 98 %, 98.2 %, 97.5 %). The 10-year OS was 95.5 %, being 98.2 %, 97.5 %, 93.8 % in pT1a, b, c. The 5- and 10-year OSs were not significantly different among the 3 different cohorts, being 97.3 % in ER+ group, 100 % (5-year) and 95.2 % (10-year) in the TN cohort, 96.6 % in Her-2+ subgroup (*p* = 0.99).

## Discussion

Breast cancer represents a heterogeneous group of tumors with different morphologic and biological features, behavior, and response to treatments. The clinical management relies on the availability of prognostic and predictive factors to support decision making, and patients are usually stratified into risk groups based on a combination of classical variables such as staging (including tumor size and nodal status) and biological variables. These risk categories are often useful for assessing prognosis and evaluating risk in groups of patients, even if their role in individual patient is more limited, and recently, various molecular techniques, particular a gene expression profiling, have been increasingly employed to assess prognosis and response to treatment. International guidelines clearly recommend adjuvant systemic treatment for node-negative breast tumors larger than 1 cm, particularly in Her-2 positive or triple negative subgroups, but definite indication for smaller tumors are lacking (NCCN 2012). Some retrospective studies of outcome for patients with T1a, b, node-negative breast cancer, treated with local therapy and commonly without adjuvant treatments, reported a very favorable 10-year RFS (>90 %), and on these basis, many clinicians might not recommend systemic treatment in view of the small additional benefit balanced against costs and toxicity (Banerjee and Smith 2010). Indeed, other retrospective evaluations highlighted the poor prognosis in some subsets of small cancers: a series from the MD Anderson Cancer Center, including 965 patients with small node-negative breast cancers not receiving adjuvant chemotherapy or trastuzumab, showed a higher risk of recurrence in Her-2 positive and TN subsets (Gonzalez-Angulo et al. 2009). A survey from Milan included 150 pT1ab node-negative, Her-2 positive breast cancer and showed a low risk of recurrence at 5 years, but, in patients ER positive, Her-2 overexpression was associated with a worse relapse-free survival (DFS) (Curigliano et al. 2009). Another recent evaluation concerning the risk of recurrence for small node-negative breast cancer by age and tumor subtypes showed that, after adjusting for subtype and other tumor characteristics, patients ≤35 years had greater risk of worse RFS compared to patients older than 50 years and confirmed the unfavorable outcome of Her-2 positive and triple negative T1a and b tumors (Theriault et al. 2011). A worse outcome for TN node-negative pT1a, b breast cancer was reported in three other retrospective evaluations on small series of 47 (Lai et al. 2011), 110 (Kaplan et al. 2009), and 56 patients (Kwon et al. 2010), respectively. In a recent small French series, 75 patients with pT1a, b, node-negative, Her-2-positive tumors were identified, and 44 % received chemotherapy and trastuzumab; even if numbers are very small, recurrences occurred only in the group receiving hormonal treatment and not receiving chemotherapy and trastuzumab (Rodrigues et al. 2010 **).** A retrospective, single institution, sequential cohort study of node-negative, small (≤2 cm), Her-2 positive patients showed that trastuzumab-treated patients derived significant benefit in RFS and OS in comparison with the no-trastuzumab-treated patients (McArthur et al. 2011). A further analysis on 267 node-negative, 1 cm or smaller tumors, revealed that patients with Her-2 positive, (particularly 0.5–1.0 cm) have a significant recurrence risk if not treated (Horio et al. 2012). In contrast, a recent evaluation on 194 node-negative pT1a, b breast cancer, 58 % treated with adjuvant chemotherapy, showed an excellent 5-year outcome irrespective of chemotherapy (Ho et al. 2012). A recent report on 2011 San Gallen Consensus Conference recommended the use of trastuzumab for Her-2 positive tumors between 5 and 10 mm, usually with chemotherapy, while no clear indication was given for triple negative tumors smaller than 1 cm (Goldhirsch et al. 2011; Gnant et al. 2011).

A recent review on trials examining the prognostic value of Her-2 in T1a, b, node-negative breast cancers confirmed the worse outcome for Her-2 positive cohort in some but not all the studies, and in some instances, number needed to treat to save one patient from recurrence was higher than number needed to harm for adverse cardiac event (Kelly et al. 2011 **),** as a confirmation of the controversies still existent on adjuvant decision making concerning small tumors.

The results of our study appear to suggest the need of systemic treatment even in pT1b, node-negative breast tumors, in Her-2 positive or triple negative subtypes, as evidenced by the slightly higher recurrence rate in pT1b than pT1c tumors, possibly related to the higher percentage of chemotherapy-treated patients in pT1c stage; these results are in line with those of Park et al. data, suggesting the use of systemic chemotherapy and Her-2 directed therapy for pT1b, node-negative Her-2 positive and chemotherapy for pT1b triple negative subgroups (Park et al. 2010); these data are also confirmed by another experience in 164 node-negative, Her-2 positive small tumors showing similar low disease recurrence risk for pT1a, pT1b and pT1c, possibly due to more frequent use of adjuvant chemotherapy in pT1c tumors; moreover, recurrence rate in pT1a, b was lower in chemotherapy-treated patients (4 vs. 17 %), (Black et al. 2006).

Other tumor characteristics are certainly involved in prognosis definition, even in very small tumors, such as histologic grading, proliferative index, or young age. As for as histologic grade concerns, some evidences indicate that tumors ≤1 cm, grade 3, have higher risk of relapse without adjuvant systemic treatment (Chia et al. 2004) or with treatments in a small percentage of patients (Hanrahan et al. 2007); our data are in line with these results, resulting tumor grade an independent prognostic factor at Cox multivariate analysis. Tumor proliferation status was another relevant prognostic factor evaluated in the literature series in small, node-negative, breast cancers (Hanrahan et al. 2007; Colleoni et al. 2004; Railo et al. 2007), resulting a high proliferative index a significant negative prognostic factor; in our casistic, Ki-67 was confirmed to be an independent prognostic factor at multivariate Cox analysis. As young age at diagnosis concerns, it was frequently associated with a higher rate of recurrence (Kwon et al. 2010; Livi et al. 2012); in our series, only 18 patients were younger than 35 years, and as expected, this issue resulted frequently related with Her-2 positivity or triple negative status, but the limited sample size does not allow a correct analysis.

Several limitations of the present study should be considered when interpreting results; it is a multicentric and retrospective evaluation, with heterogeneity in pathology teams, systemic treatments, and lacking of some data, but length of follow-up period is satisfactory. In fact, it must be considered that many of the published trials on node-negative small tumors reporting very favorable RFS have small sample size, or too short follow-up; studies with longer follow-up periods, reporting 10, 15, 20-year RFS, suggest a continued risk of late relapse (Black et al. 2006; Ichizawa et al. 2002; Quiet et al. 1995).

The clinical management of breast cancer relies on the availability of robust prognostic and predictive factors to support decision making, and stratification into risk groups is based on a combination of classical variables (staging) including tumor size and biological variables. Additional prognostic markers are needed to tailor adjuvant systemic treatment particularly for patients at “low-risk”, such as small, node-negative cancers. Recently, the metastatic potential of pT1 breast cancer was evaluated by the 70-gene MammaPrint signature in 964 early breast cancer patients, resulting the signature an independent prognostic factor (Mook et al. 2010). Certainly, the gene expression profiling technology has revolutionized breast cancer research and has been widely expected to improve prognosis, and hopefully, it will be largely available in clinical setting, complementing traditional methods with additional information, particularly in indeterminate risk group, such as node-negative, small breast cancers. Awaiting the largely availability of molecular technologies, the use of classical prognostic and predictive parameters may still allow a precise definition of the real recurrence risk in different tumor subsets.

In conclusion, the present study confirms that the prognosis of patients with node-negative small breast cancer depends on variable features. Although the number of recurrence was low in our experience, related to the fact that we are examining a population with low intrinsic metastatic potential, the observation of a worse outcome in a subset of very small breast cancer of specific unfavorable subtypes suggested that an appropriate systemic adjuvant treatment should be considered also for pT1b, Her-2 positive or triple negative breast cancer patients, even if the incremental gain in absolute benefit cannot be actually predicted, and long-term toxicities should be carefully considered on an individual basis during the decision making process.
